# Interactions between Endoplasmic Reticulum Stress and Autophagy: Implications for Apoptosis and Neuroplasticity-Related Proteins in Palmitic Acid-Treated Prefrontal Cells

**DOI:** 10.1155/2021/8851327

**Published:** 2021-10-04

**Authors:** Xiangli Xue, Feng Li, Ming Cai, Jingyun Hu, Qian Wang, Shujie Lou

**Affiliations:** ^1^Key Laboratory of Exercise and Health Sciences of Ministry of Education, Shanghai University of Sport, Shanghai, China; ^2^Shanghai Frontiers Science Research Base of Exercise and Metabolic Health, China; ^3^College of Physical Education, Guangxi University of Science and Technology, Liuzhou, China; ^4^College of Rehabilitation Sciences, Shanghai University of Medicine & Health Sciences, Shanghai, China

## Abstract

Lipotoxicity of palmitic acid (PA) or high-fat diets has been reported to increase endoplasmic reticulum (ER) stress and autophagy in peripheral tissue as well as apoptotic cell death. It also can lead to an AD-like pathological pattern. However, it has been unknown that PA-induced ER stress and autophagy are involved in the regulation of neuroplastic abnormalities. Here, we investigated the roles of ER stress and autophagy in apoptosis and neuroplasticity-related protein expression in PA-treated prefrontal cells. Prefrontal cells dissected from newborn Sprague-Dawley rats were treated with PA compound with ER stress inhibitor 4-phenylbutyric acid (4-PBA) and autophagy inhibitor 3-methyladenine (3-MA) or PA alone. PA promoted ER stress and autophagy and also cause apoptosis as well as a decline in the expression of neuroplasticity-related proteins. Inhibition of ER stress decreased the expressions of neuroplasticity-related proteins and reduced autophagy activation and apoptosis in PA-treated prefrontal cells. Inhibition of autophagy exacerbated apoptosis and enhanced ER stress in PA-treated prefrontal cells. The present study illustrated that both ER stress and autophagy could be involved in apoptosis and decreased neuroplasticity-related proteins, and the interaction between ER stress and autophagy may play a critical role in apoptosis in PA-treated prefrontal cells. Our results provide new insights into the molecular mechanisms in vitro of lipotoxicity in obesity-related cognitive dysfunction.

## 1. Introduction

In the modern environment, overconsumption of foods high in calories and saturated fatty acids, especially palmitic acid (PA), leads to weight gain and hyperlipidemia and, subsequently, obesity. Studies both in a rodent model and a clinical sample of obese individuals have shown that obesity results in impaired cognitive function, including impairments in learning and memory. The prefrontal cortex has been viewed as a mediator to obesity-related emotional and cognitive dysfunction [1]. However, little has been known about the mechanisms in the prefrontal cortex for cognitive impairment associated with obesity or excessive consumption of saturated fats.

Several studies have found that impaired neural plasticity and neural apoptosis can contribute to obesity-associated cognitive decline. Brain-derived neurotrophic factor (BDNF), synaptophysin (SYN), and acetylcholine receptor (AChR) are abundantly expressed in the prefrontal cortex and contribute to neural plasticity and learning and memory. BDNF enhances synaptic connectivity through transcription and protein synthesis and has an influence on the synthesis of neurotransmitters and neurotrophic factors. BDNF knockout mice have been found deficits in basal synaptic transmission and hippocampal long-term potentiation (LTP) [2]. SYN, a presynaptic marker, is responsible for synaptic vesicle endocytosis [3]. It has been reported that SYN performs critical functions in synaptic plasticity [4]. AChR is known to serve a distinct role in modulating neuronal plasticity and synaptic transmission [5]. Therefore, the above three indicators are considered a marker of neural plasticity in the mature brain. In addition to neural plasticity, neural apoptosis has been reported to be another important mechanism for cognitive deficits induced by streptozotocin-induced diabetes and obese insulin resistance [6, 7]. As the most abundant dietary free fatty acid, PA may exert an important effect on the regulation of neuroplasticity-related protein expression and apoptosis. PA has been demonstrated to decrease BDNF mRNA levels in primary astrocytes [8]. Rat cortical cells exposed to high levels of PA can trigger a strong apoptotic response [8]. What is more, a PA-rich HFD has been found to reduce the BDNF level and the survival of newly generated cells in the hippocampal dentate gyrus [9]. Besides, evidence in vitro of cultured hepatocytes and cardiomyocytes suggests that PA may induce apoptosis. Therefore, it is necessary to target neuroplasticity and apoptosis of prefrontal cells to elucidate the mechanism of obesity-induced cognitive impairment.

It has been reported that PA-induced lipotoxicity is linked to a series of cellular processes, such as ER stress and autophagy, which are both involved in cell death. The unfolded protein response is an adaptive response to ER stress that first attempts to alleviate its stress by reducing ER protein load and improving folding capacity and clearance of misfolded proteins but shifts toward apoptosis under prolonged and severe ER stress. PA treatment and a palmitic acid-enriched diet both have been shown to induce ER stress in SH-SY5Y human neuroblastoma cells and mouse brain, respectively [10]. Moreover, ER stress has been proved to play a central role in palmitic acid lipotoxicity-mediated apoptosis in primary rat hepatocytes [11]. It has been reported that PA induces apoptosis through overactivation of ER stress pathways in human renal proximal tubule cells [12]. Likewise, rats after exposure to intense exercise have been found hippocampus apoptosis and synapse plasticity damage through ER stress [13]. However, the relations of ER stress and apoptosis and impaired neuroplasticity in PA-treated prefrontal cells remain unclear.

As one of the stress-adaptation pathways, autophagy participates in the removal of damaged organelles and proteins and thus plays an important role in controlling the process of cell survival and apoptosis [14]. It has been suggested that inhibition of autophagy by 3-MA enhances apoptosis in EC9706 cancer cells [15]. Beclin-1 is required for the initiation of the formation of the autophagosome and may interact with antiapoptotic Bcl-2 and thus is an important convergence point of autophagy and apoptosis [16]. During the formation of autophagosome, the soluble form of LC3 (LC3-I) is converted to the autophagic vesicle-associated form (LC3-II), so the ratio of LC3-II to LC3-I could reflect the level of autophagy [17]. Recent findings have shown that the modulation of p62 by autophagy is a key factor in apoptosis and thus plays a critical role in controlling cell death and survival. PA has been proved to cause an increase in autophagic flux in cultured mouse embryonic fibroblasts and HepG2 cells [15], and inhibition of autophagy may sensitize the cells to PA-induced apoptosis, suggesting the prosurvival function of autophagy induced by PA. From our previous observations in vivo, high-fat diets-induced obesity in rats triggered excessive ER stress and apoptosis and decreased neuroplasticity-related proteins in the prefrontal cortex [18], while a causal molecular signaling mechanism is needed to be established. The potential protective effect of autophagy on PA-induced apoptosis and impaired neuroplasticity in prefrontal cells needed to be investigated.

Some studies have demonstrated the interactions between ER stress and autophagy in response to PA in peripheral tissues, but this relationship in the central nervous system needs to be further determined. On the one hand, it is likely that autophagy is engaged to defend cells against persistent ER stress and maintain ER homeostasis. Autophagy acts as stress adaptation and contributes to removing the overload of unfolded or misfolded protein that exceeds the ER capacity. On the other hand, there may be several biochemical pathways in which ER stress regulates autophagy. PERK and IRE1 are considered distinct initiators of ER stress-triggered autophagy [19]. ER stress promotes the separation of Beclin-1 and Bcl-2 and induces LC3 conversion to LC3-II to enhance autophagy activity via the activation of the PERK-eIF2*α* pathway [20, 21]. IRE1 induces Bcl-2 phosphorylation mediated by JNK leading to its dissociation with Beclin-1 resulting in activation of autophagy [22]. The eIF2*α*/ATF4/CHOP pathway closely regulates the autophagy-related gene transcriptional program in response to ER stress [23]. Thus, the crosstalk between ER stress and autophagy may provide a new therapeutic strategy against PA-induced toxicity in the brain.

In this study, we examined the effect of PA on the expressions of apoptosis markers and neuroplasticity-related proteins in prefrontal cells and the roles of ER stress and autophagy in apoptosis and disrupted neuroplasticity in PA-treated prefrontal cells. We also clarified whether there is an interplay between ER stress and autophagy in regulating apoptosis, with subsequent impairment of neuroplasticity in PA-treated prefrontal cells, which may provide a novel perspective on establishing a theoretical basis in vitro of obesity-related cognitive decline.

## 2. Materials and Methods

### 2.1. Antibodies and Reagents

Antibodies to GRP78, IRE1*α*, PERK, eIF2*α*, ATF4, Bcl-2, Bax, LC3II, LC3I, p62, and phospho-specific antibodies to p-IRE1*α*/p-PERK p-eIF2*α* and p-JNK were from Cell Signaling Technology (Beverly, MA, USA). Anticaspase 9, CHOP, BDNF, SYN, and AChR antibodies were obtained from Abcam Inc. (Cambridge, MA, USA). Anti-Beclin-1 antibody was purchased from Novus Biologicals (Littleton, San Diego, USA). Anti-FATP1 antibody was obtained from Santa Cruz Biotechnology (Dallas, Texas, CA, USA). Palmitic acid (PA) and 4-phenylbutyrate (4-PBA) were from Sigma-Aldrich Corp. (St. Louis, MO, USA). 3-Methyladenine (3-MA) was purchased from MedChemExpress (HY-19312, New Jersey, USA).

### 2.2. Cell Culture and Treatment

Newborn Sprague-Dawley (SD) rats were purchased from the Shanghai Lab Animal Center (Certificate SCXK 2013–0016). Rats were soaked in 75% ethanol for disinfection and then were sacrificed. The brains were removed and soaked in D-Hank's solution. The prefrontal cortex was dissected from the brain. After carefully removing the meninges and blood vessels, the prefrontal cortex was minced and digested with 0.25% trypsin for 10-15 min at 37°C. Then, the digestion procedure was terminated by adding Dulbecco's modified Eagle's medium (DMEM, Gibco) supplemented with 10% (*v*/*v*) fetal bovine serum (FBS, Gibco) and 1% penicillin-streptomycin (Beyotime, Shanghai, China). The liquid was pipetted into suspension with a straw repeatedly and filtered through a 200-mesh sieve hole; the filtered suspension was seeded in 6-well plates which were coated with poly-L-lysine (Sigma-Aldrich), incubated with the medium in a humidified incubator at 37°C and 5% CO_2_. The culture medium was refreshed every 2-3 days.

Palmitate acid (PA), 4-phenylbutyrate (4-PBA), and 3-methyladenine (3-MA) were dissolved in DMSO. After culturing for 5 days, prefrontal cells were treated with different concentrations of PA (0-1.0 mM) for 24 h and harvested, and the apoptosis was analyzed by flow cytometry. After treatment with palmitate acid, cells were stimulated with different concentrations of 4-PBA (5, 7.5, and 10 mM) and 3-MA (2.5, 5, and 10 mM) for 24 h. According to the preexperiment, 5 mM 4-PBA inhibitor and 2.5 mM 3-MA inhibitor were finally chosen for the follow-up experiments. The same concentration of neurobasal medium was served as the control. Cells were collected and processed for further experiments.

### 2.3. Flow Cytometry Analysis of Cell Apoptosis

Primary prefrontal cells were trypsinized, rinsed, and harvested. Flow cytometry analyses were performed using the Annexin V-FITC apoptosis detection kit (Selleck Chemicals, Houston, TX, USA) according to the manufacturer's instructions. The cells were pelleted by centrifugation and resuspended followed by being stained with Annexin V-FITC and PI for 10 min in the dark at room temperature. The cells were finally analyzed by using an FC500 System flow cytometer (Becton-Dickinson, Franklin, NJ, USA).

### 2.4. Western Blot

After treatment with the indicated conditions, primary prefrontal cells were washed with ice-cold PBS twice and collected. Ice-cold Membrane Nuclear and Cytoplasmic Protein Extraction kit (No. C510002, Sangon Biotech, Shanghai, China) was used to extract membrane, cytoplasmic, and nuclear proteins following the manufacturer's instructions. The protein concentration was measured by the BCA method (BCA Protein Assay Kit, P0010, Beyotime, Shanghai, China), followed by boiling in the gel-loading buffer for 5 minutes at 100°C. Equal amounts of proteins were separated by SDS-PAGE gel, transferred to a nitrocellulose membrane, and incubated with specific primary antibodies overnight. After washing with TBST 4 times for 8 min each, the membranes were incubated with HRP-conjugated secondary antibodies for 2 hours at room temperature, and bands were detected using an enhanced chemiluminescence (ECL) kit. The density of the bands was measured quantitatively using ImageJ.

### 2.5. Real-Time PCR

Total RNA was extracted from primary prefrontal cells cultured in six-well plates by using the TRIzol reagent (Invitrogen, Chromos, Singapore) as instructed by the manufacturer. 2 *μ*g of total RNA was converted into cDNA using the ReverTra Ace® qPCR RT Kit (TOYOBO, Osaka, Japan). Real-time PCR was performed with the SYBR Green real-time PCR kit (TOYOBO) in StepOnePlus™ Real-Time PCR System (Applied Biosystems, CA, USA). A reaction system with its final volume of 20 *μ*l was made with 2.0 *μ*l template, 4 *μ*mol of each primer, and 2.0x Master SYBR Green I. The reactions were incubated at 95°C for 5 min (holding stage) followed by 40 cycles of denaturation at 95°C for 15 seconds, annealing at 55°C for 45 seconds, and extension at 72°C for 45 seconds. The primer dimer in the PCR products was determined by melting curve analysis after the PCR amplification. The relative levels of interest genes were normalized by *β*-actin as an internal control and calculated using the 2^-*ΔΔ*Ct^ method. PCR primers were as follows: FATP1 (sense 5′-GCAGCATTGCCAACATGGAC-3′ and antisense 5′-GTGTCCTCATTGACCTTGACCAGA-3′), SYN (sense 5′-CTGCGTTAAAGGGGGCACTA-3′ and antisense 5′-ACAGCCACGGTGACAAAGAA-3′), BDNF (sense 5′-AACCATAAGGACGCGGACTT-3′ and antisense 5′-TGCAGTCTTTTTATCTGCCG-3′), AChR (sense 5′-TGCAAAGAGCCATACCCAGA-3′ and antisense 5′-TGATCCTGGTCCACTTAGGC-3′), and *β*-actin (sense 5′-AGAAGCTGTGCTATGTTGCTCTA-3′ and antisense 5′-TCAGGCAGCTCATAGCTCTTC-3′).

### 2.6. Statistical Analyses

The data are presented as the mean ± SEM. Statistical analyses were performed using the SPSS software package (version 20.0; SPSS, Chicago, IL, USA). Data were analyzed using an independent sample *t*-test or one-way ANOVA with Tukey's HSD test to evaluate intergroup differences. All experiments were repeated at least three times for each group. *p* < 0.05 was considered statistically significant.

## 3. Results

### 3.1. The Dose-Response Effect of PA on Apoptosis in Prefrontal Cells

**Figure 1:**
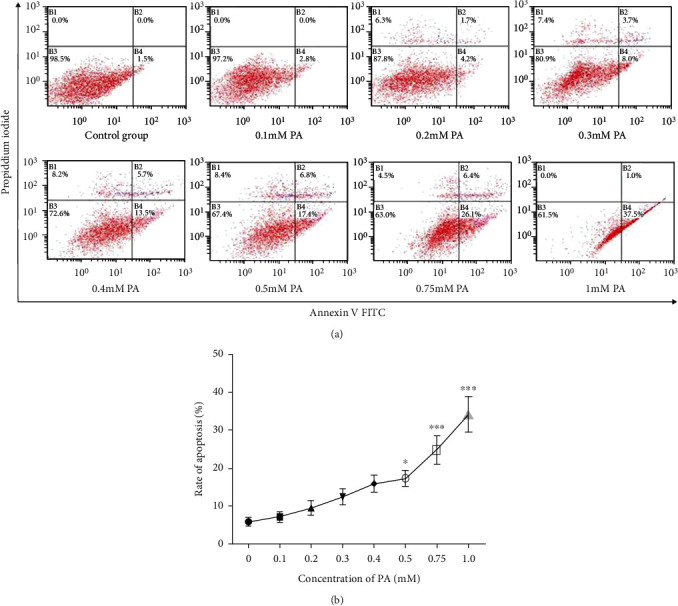
Prefrontal cells were treated with 0.1, 0.2, 0.3, 0.4, 0.5, 0.75, and 1.0 mM PA for 24 h. Cells were subjected to the flow cytometry assay, and representative results are shown (a). The rate of apoptosis in each group was calculated based on Annexin V and PI staining assays (b). Data are presented as means ± SEM. ^∗^*p* < 0.05 and ^∗∗∗^*p* < 0.001 versus control (Con).

To observe the effect of PA on apoptosis, prefrontal cells were exposed to increasing concentrations of PA, and the incidence of apoptosis was measured by flow cytometry. As shown in Figure 1, PA significantly increased the apoptosis rate of prefrontal cells at doses of 0.5 mM, 0.75 mM, and 1.0 mM. The median apoptotic rate of the 0.5 mM PA group was 17.23 ± 3.02 (*p* < 0.05), and the apoptosis rate of the 1.0 mM PA group was the highest, reaching 34.15 ± 7.51 (*p* < 0.01).

### 3.2. Effects of 4-PBA and 3-MA on the Expression of FATP1 in PA-Treated Prefrontal Cells

**Figure 2:**
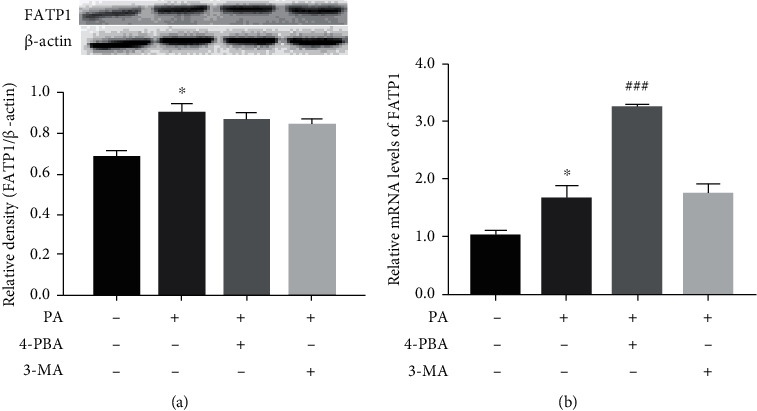
Effects of 4-PBA and 3-MA on the expression of FATP1 in PA-treated prefrontal cells. The protein and mRNA levels of FATP1 were detected by Western blot (a) and RT-PCR (b) at 24 h after treatment with 4-PBA (5 mM) and 3-MA (2.5 mM) compound with PA (0.5 mM) or PA alone, and *β*-actin was used as an internal control. The results were representative of at least three independent experiments. Data are presented as means ± SEM. ^∗^*p* < 0.05 versus control (Con).

To determine whether the expression of FATP1 is regulated by palmitic acid, ER stress, and autophagy, prefrontal cells were treated with 4-PBA (5 mM) and 3-MA (2.5 mM) compound with PA (0.5 mM) or PA alone; FATP1 mRNA and protein level were detected by RT-PCR and Western blot, respectively. As shown in Figure 2, PA treatment markedly increased the mRNA and protein level of FATP1 (*p* < 0.01), compared to the control. However, compared with the PA group, there was no significant difference in FATP1 protein expression both in the 4-PBA and 3-MA groups. An increased mRNA expression of FATP1 was observed in the 4-PBA group compared with the PA group.

### 3.3. Effects of 4-PBA and 3-MA on the Expression of ER Stress Markers in PA-Treated Prefrontal Cells

**Figure 3:**
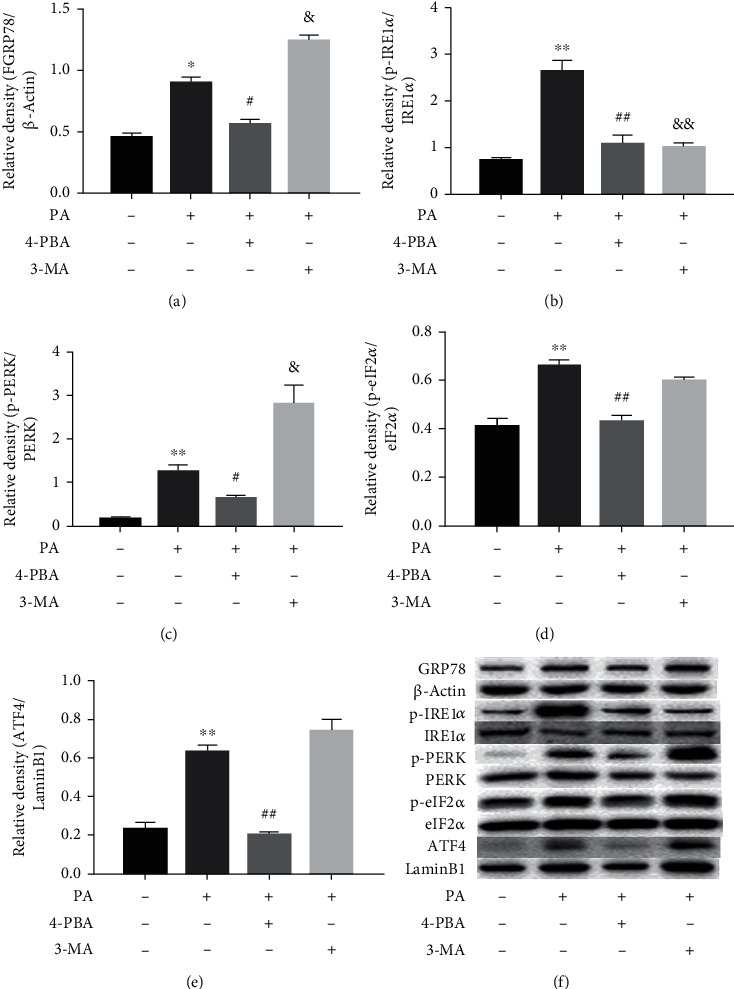
Effects of 4-PBA and 3-MA on the expression of ER stress markers in PA-treated prefrontal cells. The relative protein levels of GRP78 (a), p-IRE1*α* (b), p-PERK (c), p-eIF2*α* (d), and ATF4 (e) in prefrontal cells, and a representative Western blot image was shown (f). Data are presented as means ± SEM. ^∗^*p* < 0.05 and ^∗∗^*p* < 0.01 versus control (Con); ^#^*p* < 0.05, ^##^*p* < 0.01, and ^&^*p* < 0.05 versus PA.

To further clarify the roles of ER stress and autophagy in the levels of ER stress markers in PA-treated prefrontal cells, cells were treated with 4-PBA (5 mM) and 3-MA (2.5 mM) compound with PA (0.5 mM) or PA alone. ER stress-related proteins, including GRP78, p-IRE1*α*, p-PERK, p-eIF2*α*, and ATF4, were detected by Western blot. PA significantly increased levels of GRP78 (*p* < 0.05), p-IRE1*α* (*p* < 0.01), p-PERK (*p* < 0.01), p-eIF2*α* (*p* < 0.01), and ATF4 (*p* < 0.01). ER stress inhibitor 4-PBA restored protein levels of GRP78 (*p* < 0.05), p-IRE1*α* (*p* < 0.01), p-PERK (*p* < 0.05), p-eIF2*α* (*p* < 0.01), and ATF4 (*p* < 0.01) in PA-treated prefrontal cells. Autophagy inhibitor 3-MA also restored protein levels of GRP78 (*p* < 0.05), p-IRE1*α* (*p* < 0.01), and p-PERK (*p* < 0.05) in PA-treated prefrontal cells. However, there was no significant difference in p-eIF2*α* and ATF4 expression between the PA group and the PA cotreatment with autophagy inhibitor 3-MA group, as shown in Figure 3.

### 3.4. Effects of 4-PBA and 3-MA on the Expression of Autophagy Markers in PA-Treated Prefrontal Cells

**Figure 4:**
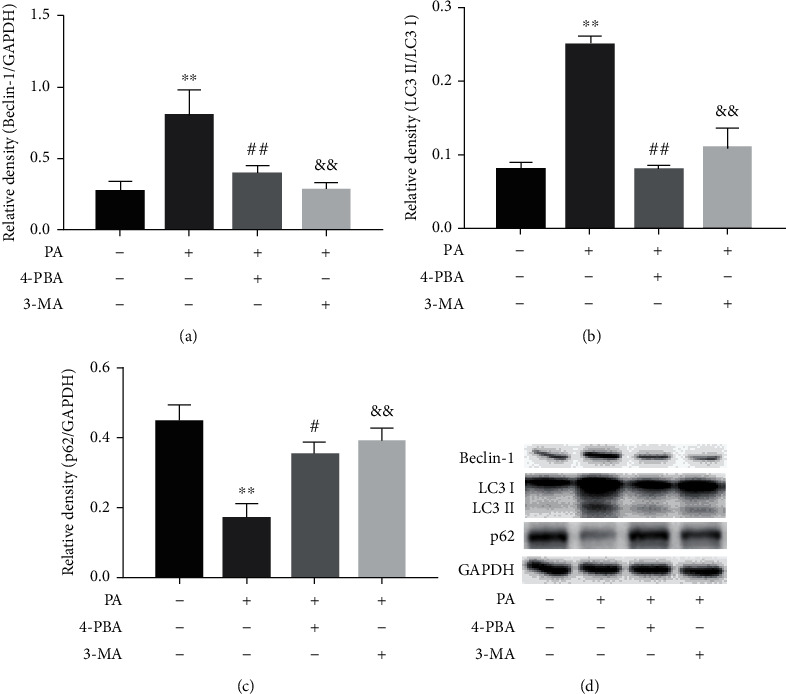
Effects of 4-PBA and 3-MA on the expression of autophagy markers in PA-treated prefrontal cells. The protein levels of autophagy-related proteins, including Beclin-1 (a), LC3 (b), and p62 (c) in prefrontal cells, and a representative Western blot image was shown (d). Data are presented as means ± SEM. ^∗∗^*p* < 0.01 versus control (Con); ^#^*p* < 0.05, ^##^*p* < 0.01, and ^&&^*p* < 0.01 versus PA.

To further assess the roles of ER stress and autophagy in the levels of autophagy markers in PA-treated prefrontal cells, cells were treated with 4-PBA (5 mM) and 3-MA (2.5 mM) compound with PA (0.5 mM) or PA alone. The initiation and elongation processes of autophagosomes were examined with LC3 and Beclin-1, and the ability of autophagic clearance was measured with p62. PA significantly increased levels of Beclin-1 (*p* < 0.01) and the ratio of LC3-II to LC3-I (*p* < 0.01) as well as decreased p62 protein expression (*p* < 0.01). This effect was significantly reversed by the ER stress inhibitor 4-PBA and the autophagy inhibitor 3-MA, as shown in Figure 4.

### 3.5. Effects of 4-PBA and 3-MA on the Expression of Apoptosis Markers and Apoptosis Rate of Prefrontal Cells Treated with PA

**Figure 5:**
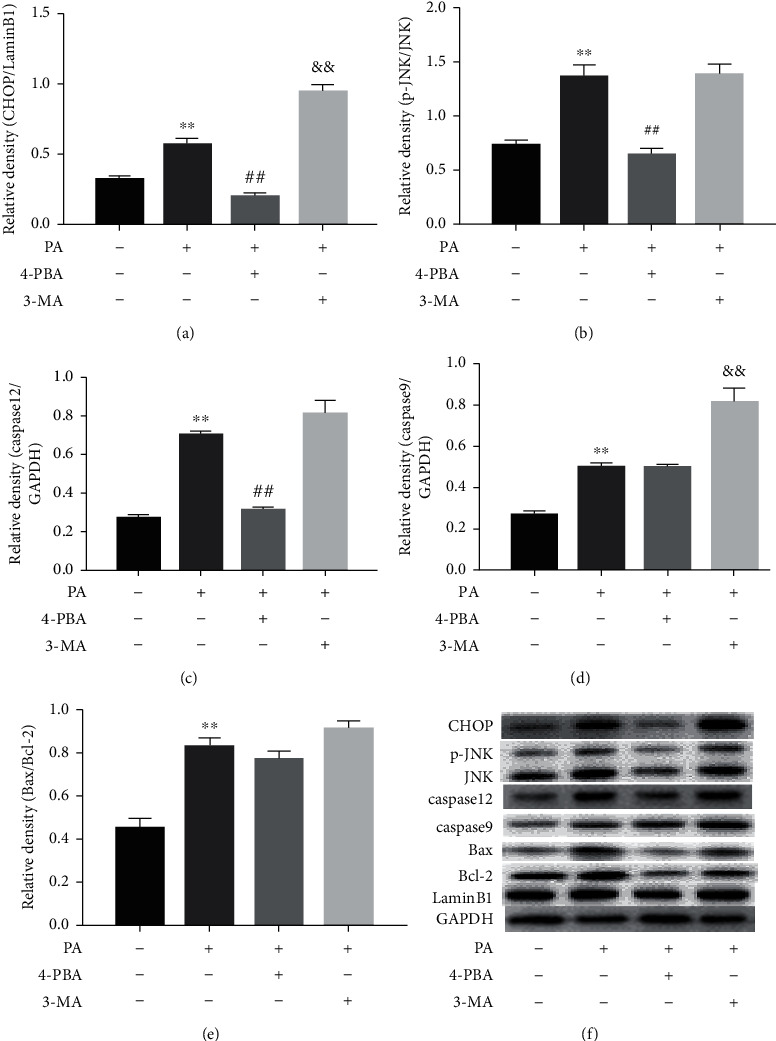
Effects of 4-PBA and 3-MA on the expression of apoptosis markers in PA-treated prefrontal cells. The protein levels of apoptosis proteins, including CHOP (a), p-JNK (b), caspase 12 (c), caspase 9 (d), and Bax/Bcl-2 (e) in prefrontal cells, and a representative Western blot image was shown (f). Data are presented as means ± SEM. ^∗∗^*p* < 0.01 versus control (Con); ^##^*p* < 0.01 and ^&&^*p* < 0.01 versus PA.

To further explore the roles of ER stress and autophagy in the levels of apoptosis markers in PA-treated prefrontal cells, cells were treated with 4-PBA (5 mM) and 3-MA (2.5 mM) compound with PA (0.5 mM) or PA alone. Apoptosis markers, including CHOP, p-JNK, caspase 12, caspase 9, and Bax/Bcl-2, were detected by Western blot. As shown in Figure 5, PA increased CHOP, caspase 12, and caspase 9 protein levels (*p* < 0.01) and phosphorylation level of JNK (*p* < 0.01), as well as the Bax/Bcl-2 ratio (*p* < 0.01), compared to the control. Compared with the 0.5 mM PA group, the protein expression of CHOP, p-JNK, and caspase 12 in the 4-PBA group was significantly decreased (*p* < 0.01), while no change was found for caspase 9 and Bax/Bcl-2 ratio. The results also showed that combination treatment using PA and 3-MA upregulated the levels of CHOP and caspase 9 more than that of PA treatment alone (*p* < 0.01). No significant differences in protein levels of p-JNK, caspase 12, or Bax/Bcl-2 ratio were observed between the PA group and the 3-MA compound with the PA group.

**Figure 6:**
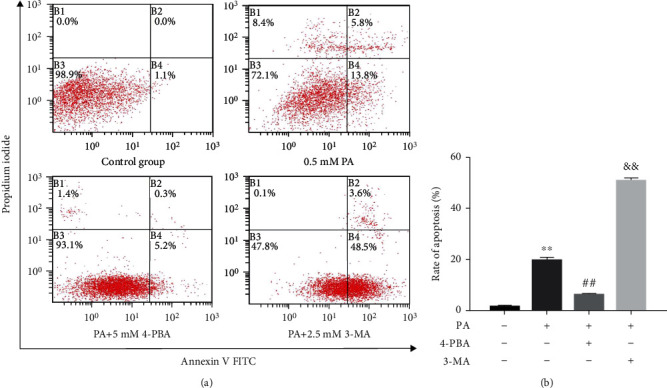
Effects of 4-PBA and 3-MA on apoptosis rate of prefrontal cells treated with PA. Prefrontal cells were treated with 4-PBA (5 mM) and 3-MA (2.5 mM) compound with PA (0.5 mM) or PA alone. (a) Cells were subjected to flow cytometry assay, and representative results are shown. (b) The rate of apoptosis in each group was calculated based on Annexin V and PI staining assays. Data are presented as means ± SEM. ^∗∗^*p* < 0.01 versus control (Con); ^##^*p* < 0.01 and ^&&^*p* < 0.01 versus PA.

For quantifying the role of ER stress and autophagy in the apoptosis rate of prefrontal cells treated with PA, 4-PBA (5 mM) and 3-MA (2.5 mM) were used to inhibit ER stress and autophagy in cells treated with 0.5 mM PA, respectively. Then, the cells were cultured for another 24 h. As shown in Figure 6, PA treatment markedly increased the rate of apoptosis (*p* < 0.01). Compared with the PA group, 5 mM 4-PBA markedly decreased the rate of apoptosis (*p* < 0.01), while 2.5 mM 3-MA markedly increased the rate of apoptosis (*p* < 0.01). These results suggested that ER stress mediates PA-induced apoptosis, while autophagy may play a protective role in PA-induced apoptosis.

### 3.6. Effects of 4-PBA and 3-MA on the Expression of Neuroplasticity-Related Proteins in PA-Treated Prefrontal Cells

**Figure 7:**
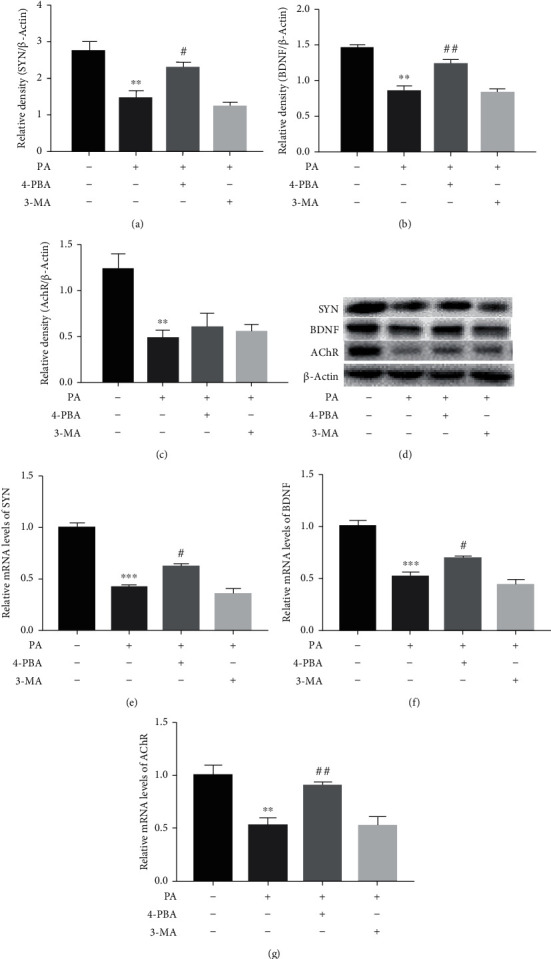
Effects of 4-PBA and 3-MA on the expression of neuroplasticity-related proteins in PA-treated prefrontal cells. The relative protein levels of SYN (a), BDNF (b), and AChR (c) in prefrontal cells, and a representative Western blot image was shown (d). The relative mRNA expression levels of SYN (e), BDNF (f), and AChR (g) in prefrontal cells. Data are presented as means ± SEM. ^∗∗^*p* < 0.01 and ^∗∗∗^*p* < 0.001 versus control (Con); ^#^*p* < 0.05 and ^##^*p* < 0.01 versus PA.

To further explore the roles of ER stress and autophagy in the expression of neuroplasticity-related proteins in PA-treated prefrontal cells, cells were treated with 4-PBA (5 mM) and 3-MA (2.5 mM) compound with PA (0.5 mM) or PA alone. The expression of mRNA and proteins involved in neural plasticity such as SYN, BDNF, and AChR was detected by RT-PCR and Western blot, respectively. PA significantly decreased expression of SYN (*p* < 0.01 at the protein level and *p* < 0.001 at the mRNA level), BDNF (*p* < 0.01 at the protein level and *p* < 0.001 at the mRNA level), and AChR (*p* < 0.01 at the protein and mRNA levels). ER stress inhibitor 4-PBA restored protein levels of SYN (*p* < 0.05) and BDNF (*p* < 0.01)and reversed the decreased mRNA levels of SYN (*p* < 0.05), BDNF (*p* < 0.05), and AChR (*p* < 0.01) in PA-treated prefrontal cells. There was no significant difference in all three neuroplasticity-related protein expressions between the PA group and the PA cotreatment with autophagy inhibitor 3-MA group, as shown in Figure 7.

## 4. Discussion

Our previous studies have suggested that high-fat diet-induced obesity upregulates the expression of FATP1 in the prefrontal cortex and hippocampus and downregulates the expression of neuroplasticity-related proteins [18, 24]. It is well known that one of the major features of obesity is the increase in circulating fatty acid (FA) serum levels. Since fatty acid oxidation is rarely used to supply energy by the brain, the FA content in brain tissue is very small under physiological conditions. However, in the long-term high-fat environment, there is a compensatory increase in FATP1 on the cell membrane that serves excessive fatty acid transport into the brain and thereby causes lipid toxicity by a disorder of intracellular lipid metabolism [25, 26]. PA is considered the most common saturated fatty acid in food. In in vitro experiments, PA can imitate the lipid toxicity caused by a long-term high-fat diet, and intracellular accumulation of excessive levels of PA can cause lipotoxicity and neurotoxicity. However, the detailed mechanisms are not as well understood. In this study, we explored the mechanisms of lipotoxicity by using PA to treat prefrontal cells.

Accumulating evidences have demonstrated that PA impairs neuroplasticity and triggers apoptosis. In the present study, we demonstrated that PA increased the expression of FATP1 and the apoptosis rate in prefrontal cells and decreased the protein and mRNA expression of SYN, BDNF, and AChR. This suggests that PA-induced lipotoxicity causes cell death and downregulates the expression of neuroplasticity-related proteins. Consistent with our findings, HFD has been reported to increase PA deposition in the hippocampus and inhibit synaptic plasticity, at least partly, contributing to impairment in hippocampal-dependent memory [27]. Both 48 h high-fat diets and in vitro treatment with PA have been reported to impair long-term potentiation in hippocampal slices [27]. In the prefrontal cortical neurons, PA has been found to impair leptin-mediated presynaptic marker SYN and postsynaptic density marker 95 immunoreactivity as well as reduce the mRNA expression of BDNF [28]. We also demonstrated that 4-PBA or 3-MA did not restore protein expression of FATP1 in PA-treated prefrontal cells, suggesting that FATP1 protein expression may be dependent on the PA rather than ER stress or autophagy.

Next, in order to determine the impact of PA on ER stress, rat prefrontal cells were treated with PA, which significantly increased ER stress markers GRP78, p-IRE*α*, p-PERK, p-eIF2*α*, and ATF4, and also cause a decline in the expression of neuroplasticity-related proteins. ER stress inhibitor 4-PBA restored protein levels of SYN and BDNF in PA-treated prefrontal cells. As we all know, the endoplasmic reticulum is the major site of protein synthesis, posttranslational modification and transportation, and lipid metabolism. Intracellular accumulation of excess fatty acids (such as PA) aggravates the burden of the ER. Therefore, PA-related pathological conditions disturb ER homeostasis and lead to ER stress, as a result of the decline in the synthesis of neural plasticity mature protein molecules (including BDNF), as well as the accumulation of unfolded or misfolded proteins in the ER lumen. Our previous studies have shown that high glucose and PA can induce excessive ER stress and apoptosis via promoting the overexpression of GLUT3 and FATP1, and ER stress can suppress BDNF and SYN expression through negatively regulating the p38/ERK-CREB pathway and positively regulating the NLRP3-IL-1*β* pathway in cultured primary hippocampal cells [29]. This may partly account for the effect of PA on the expression of plasticity-related proteins. Moreover, excessive and/or prolonged unfolded protein response activation leads to ER stress-induced apoptosis [30]. Previous studies have shown that saturated fatty acids (such as PA) cause ER stress in many peripheral tissues, including the pancreatic islets, heart, liver, and muscle, leading to cell dysfunction or death through lipotoxicity. Similarly, our results verified that these conclusions could be established in the prefrontal cells. We demonstrated PA's ability to intensify ER stress, which we hypothesized may trigger apoptosis and damage synaptic plasticity. Incidentally, in the literature, 4-PBA has been cited as anti-ER stress. In order to determine the impact of PA on ER stress, rat prefrontal cells were treated with PA, which significantly increased ER stress markers GRP78, p-IRE*α*, p-PERK, p-eIF2*α*, and ATF4. We also found that PA increased the apoptosis rate in a dose-dependent manner and enhanced apoptosis-related protein expressions. To determine the role of ER stress in apoptosis of prefrontal cells treated by PA, 4-PBA treatment of rat prefrontal cells significantly decreased ER stress markers, which was accompanied by an augmentation of CHOP, p-JNK, and caspase 12, confirming that ER stress is involved in prefrontal cell apoptosis. Similarly, in a hypothalamic neuronal cell model, palmitate has been found to induce ER stress subsequently leading to JNK phosphorylation and activation of caspase 3 [31]. Chen et al. [32] found that PA induces apoptosis via enhancing the expression levels of the ER stress markers GRP78 and CHOP in murine Leydig tumor cell line 1 cells. Yang et al. [33] demonstrated that the ER stress GRP78-CHOP pathway is involved in PA-induced H9c2 cell apoptosis, and inhibition of ER stress by 4-PBA decreased apoptosis. In another study, PA has been found to increase the ER stress marker, p-eIF2*α*, and promote cell death in SH-SY5Y human neuroblastoma cells, which may result in accumulation of beta-amyloid, and could represent a mechanism of obesity-related neurodegenerative diseases [10]. These findings suggest that ER stress may account for apoptosis and decline of neuroplasticity-related proteins induced by PA. This may have implications in understanding cellular mechanisms underlying excess PA-related brain diseases based on the close connection between neural plasticity and cognitive function.

In addition to ER stress, lipotoxicity can also regulate cell apoptosis via many other mechanisms, such as autophagy [34]. Autophagy is an evolutionarily conserved lysosomal-dependent system found in eukaryotes, which can regulate cellular protein and organelle renewal. Autophagy activity is upregulated by lipotoxicity or other pathologic stress, which contributes to regulating cell homeostasis and protein balance. Emerging data has indicated that PA modulates autophagy in hepatocytes, pancreatic *β*-cell, and kidney proximal tubular cells [35–37]. Nevertheless, it remains undetermined whether autophagy is associated with PA-induced neuronal apoptosis and decreased neuroplasticity-related proteins. An in vivo experiment has found that long-term exposition to a diet rich in PA increases the number of Bcl-2 immunopositive cells in the dentate gyrus, and it is accompanied by decreased levels of LC3 and *β*-catenin complexes in different areas of the hippocampus [38]. It has been revealed that HFD induces a reduction in the apoptotic processes and autophagic activity in the mouse brain, mechanisms related to A*β* formation. Furthermore, we have previously shown that there is an increasing trend of the expression of Beclin-1 and LC3- II/I, but not a significant effect on the prefrontal cortex of HFD rats [18]. Meanwhile, HFD has been shown to stimulate apoptosis by upregulating the expressions of apoptosis-associated proteins, including caspase 12, CHOP, and Bax/Bcl-2. Contrary to these findings, here, we clearly show that PA drastically increases levels of Beclin-1 and ratio of LC3-II to LC3-I as well as decreased p62 protein expression, which indicates PA-induced autophagy in prefrontal cells. 3-MA has been widely used as an autophagy inhibitor based on its inhibitory effect on class III PI3K activity, which is known to be essential for the induction of autophagy [39]. In the present study, inhibition of autophagy by 3-MA enhanced the effect of PA-induced apoptosis in prefrontal cells, with elevated expression of caspase 9 and CHOP as well as apoptotic rate. Interestingly, our results indicated that CHOP-dependent apoptosis is a pathway that both 4-PBA and 3-MA share in common. In agreement with our findings, PA has been reported to induce autophagy and apoptosis in various cell types, including podocytes, H9c2 cardiomyocyte, and epithelial cell line [40–42]. It is proposed that autophagy can serve either prosurvival or prodeath functions depending on different situations [43]. These shreds of evidence brought forth a novel role of autophagy in protecting against apoptosis in prefrontal cells.

Lipotoxicity leads to apoptosis and impaired neuroplasticity and is accompanied by significant alterations of ER stress and autophagy. However, whether there is a molecular interplay among these two pathways has not yet been elucidated. It is worth noting that both ER stress and autophagy play an indispensable role in maintaining protein homeostasis and cell's survival-signaling. Accumulating evidence indicates that excessive ER stress may trigger autophagy, which likely plays a compensatory role to relieve the ER stress in neurons. However, dysregulation of ER stress and autophagy results in inefficient clearance of the accumulated proteins and thus leads to apoptosis and neurodegeneration [44]. Previous studies have shown that autophagy can be activated in PA-treated INS-1 *β*-cells and that the activated autophagy plays a protective role in PA-induced apoptotic cell death, which is the possible involvement of signaling pathways related to ER stress [45]. Both 4-PBA and 3-MA have been shown to significantly reduce the proportion of cells with GFP-LC3 positive dots in PA-treated INS-1 cells. Besides, 4-PBA has been reported to reverse advanced glycation end-products- (AGEs-) induced autophagy and cell apoptosis, while inhibition of autophagy by Atg5 siRNA transfection may aggravate AGEs-induced mesangial cell apoptosis and have no effect on AGEs-induced ER stress activation [43]. Similarly, our data showed that inhibition of ER stress with 4-PBA decreased the protein expressions of Beclin-1 and LC3II/LC3I as well as increased p62 protein expression, indicating that autophagy activation and autophagic clearance were regulated by ER stress in PA-treated prefrontal cells. In addition, inhibition of autophagy with 3-MA upregulated ER stress markers including GPR78 and p-PERK, indicating that inhibition of autophagy could aggravate ER stress. Combined with the results of apoptosis, we conclude that there are biological interactions between ER stress and autophagy in contributing to apoptosis in PA-treated prefrontal cells. Of note, autophagy has been shown to engage in a complex interplay with ER stress. Autophagy is involved in removing the overload of unfolded or misfolded protein that exceeds the ER capacity, in order to defend cells against persistent ER stress and maintain ER homeostasis. Thus, autophagy may be involved in regulating the synthesis and expression of BDNF through ER stress. However, we did not found that 3-MA changed the expression of neuroplasticity-related proteins. Future studies are needed to elucidate this further.

## 5. Conclusions

**Figure 8:**
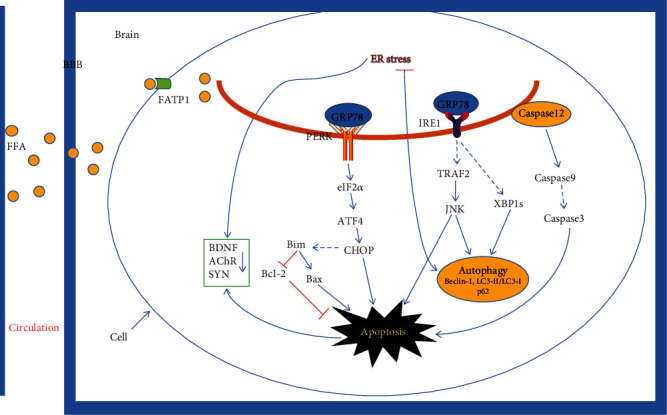
A potential schematic illustration to clearly explain the mechanism of the interactions between ER stress and autophagy in PA-treated prefrontal cell apoptosis.

In conclusion, our results demonstrate that interactions between ER stress and autophagy could play a role as a mediator for PA-induced apoptosis and the declined expression of neuroplasticity-related proteins in prefrontal cells. This may represent mechanisms of cognitive dysfunction and obesity-associated neurodegeneration in response to the long-term high-fat diets in vitro. We have drawn a potential schematic illustration referred to the previous studies and our research (showed in Figure 8). However, our study also has several limitations. LTP may be needed to be evaluated at the electrophysiological level given that LTP is one of the most representative studied forms of synaptic plasticity or neuronal plasticity.

## Data Availability

The data used to support the findings of this study are available from the corresponding author upon request.

## Supplementary Materials

Supplementary Materials Figure S1: the cytograms for the effects of PA, 4-PBA, and 3-MA. Primary prefrontal cells were, respectively, treated with 4-PBA (5 mM) or 3-MA (2.5 mM) compound with PA (0.5 mM) or PA alone for 24 h. Scale bar = 200 *μ*m. Figure S2: to explore treated concentration of 4-PBA inhibitor. (A) The effects of different concentrations of 4-PBA inhibitor on the viability rate of the prefrontal cells. (B) The effects of different concentrations of 4-PBA inhibitor on the apoptotic rate of the prefrontal cells. (C) Flow cytometry analysis was performed to measure the viability and apoptotic rate of the prefrontal cells. Data are presented as means ± SEM. ^∗^*p* < 0.05 versus control (Con). Figure S3: to explore treated concentration of 3-MA inhibitor. (A) The effects of different concentrations of 3-MA inhibitor on the viability rate of the prefrontal cells. (B) The effects of different concentrations of 3-MA inhibitor on the apoptotic rate of the prefrontal cells. (C) Flow cytometry analysis was performed to measure the viability and apoptotic rate of the prefrontal cells. Data are presented as means ± SEM. ^∗^*p* < 0.05 versus control (Con). Figure S4: effects of CQ on the expression of autophagy markers in PA-treated prefrontal cells. The protein levels of LC3 (A) in prefrontal cells, and a representative Western blot image was shown (B). Data are presented as means ± SEM. ^∗∗^*p* < 0.01 versus control (Con); ^#^*p* < 0.05 versus PA. Figure S5: effects of 4-PBA and 3-MA alone on the expression of ER stress markers in prefrontal cells. The relative protein levels of GRP78 (A), p-IRE1*α* (B), p-PERK (C), p-eIF2*α* (D), and ATF4 (E) in prefrontal cells, and a representative Western blot image was shown (F). Data are presented as means ± SEM. Figure S6: effects of 4-PBA and 3-MA alone on the expression of apoptosis markers in prefrontal cells. The protein levels of apoptosis proteins, including CHOP (A), p-JNK (B), caspase 12 (C), caspase 9 (D), and Bax/Bcl-2 (E) in prefrontal cells, and a representative Western blot image was shown (F). Data are presented as means ± SEM.
