# Supplementary material for: Interactions between Endoplasmic Reticulum Stress and Autophagy: Implications for Apoptosis and Neuroplasticity-Related Proteins in Palmitic Acid-Treated Prefrontal Cells
Source: Neural Plast. 2021 Oct 4;2021:8851327. doi: 10.1155/2021/8851327 (PMC8505096; doi:10.1155/2021/8851327)
Supplement: Supplementary Materials — Figure S1: the cytograms for the effects of PA, 4-PBA, and 3-MA. Primary prefrontal cells were, respectively, treated with 4-PBA (5 mM) or 3-MA (2.5 mM) compound with PA (0.5 mM) or PA alone for 24 h. Scale bar = 200 μm. Figure S2: to explore treated concentration of 4-PBA inhibitor. (A) The effects of different concentrations of 4-PBA inhibitor on the viability rate of the prefrontal cells. (B) The effects of different concentrations of 4-PBA inhibitor on the apoptotic rate of the prefrontal cells. (C) Flow cytometry analysis was performed to measure the viability and apoptotic rate of the prefrontal cells. Data are presented as means ± SEM. ∗p < 0.05 versus control (Con). Figure S3: to explore treated concentration of 3-MA inhibitor. (A) The effects of different concentrations of 3-MA inhibitor on the viability rate of the prefrontal cells. (B) The effects of different concentrations of 3-MA inhibitor on the apoptotic rate of the prefrontal cells. (C) Flow cytometry analysis was performed to measure the viability and apoptotic rate of the prefrontal cells. Data are presented as means ± SEM. ∗p < 0.05 versus control (Con). Figure S4: effects of CQ on the expression of autophagy markers in PA-treated prefrontal cells. The protein levels of LC3 (A) in prefrontal cells, and a representative Western blot image was shown (B). Data are presented as means ± SEM. ∗∗p < 0.01 versus control (Con); #p < 0.05 versus PA. Figure S5: effects of 4-PBA and 3-MA alone on the expression of ER stress markers in prefrontal cells. The relative protein levels of GRP78 (A), p-IRE1α (B), p-PERK (C), p-eIF2α (D), and ATF4 (E) in prefrontal cells, and a representative Western blot image was shown (F). Data are presented as means ± SEM. Figure S6: effects of 4-PBA and 3-MA alone on the expression of apoptosis markers in prefrontal cells. The protein levels of apoptosis proteins, including CHOP (A), p-JNK (B), caspase 12 (C), caspase 9 (D), and Bax/Bcl-2 (E) in prefrontal cells, and a represen [file 8851327.f1.doc]

**Supplementary data**

Primary prefrontal cells were respectively treated with 4-PBA (5 mM) and 3-MA (2.5 mM) compound with PA (0.5 mM) or PA alone, then the changes of cell morphology were observed under the microscope. Cells in control group were spindle a conical-shaped with longer protrusions, connecting the cells together in a sparse network (Fig. S1 A). Cells treatment with PA for 24h were found reduced protrusion length and cytoplasmic shrinkage, and some cells had been suspended (Fig. S1 B). Most cells kept good growth status in 4-PBA compound with PA group (Fig. S1 C), while the growth status of the cells was worse in 3-MA compound with PA group (Fig. S1 D).


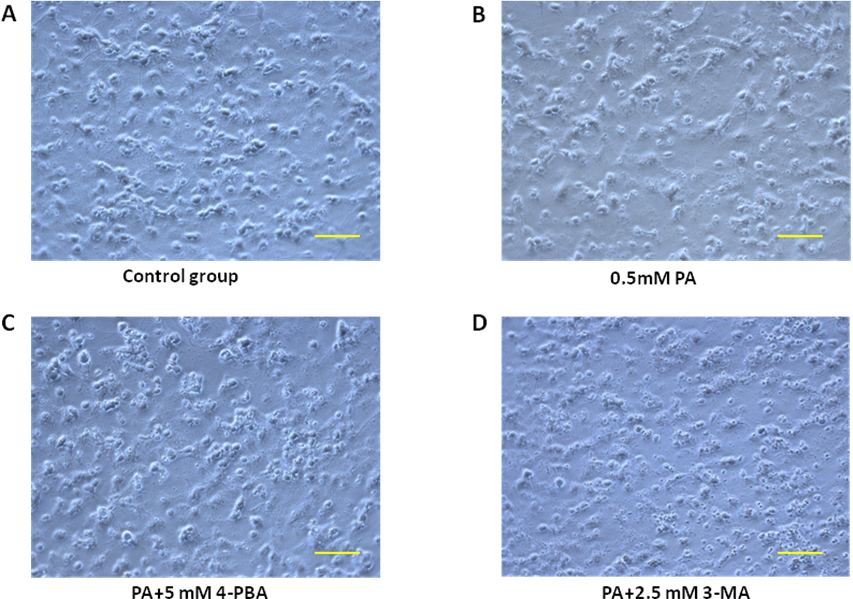


Figure S1. The cytograms for the effects of PA, 4-PBA and 3-MA. Primary prefrontal cells were respectively treated with 4-PBA (5 mM) or 3-MA (2.5 mM) compound with PA (0.5 mM) or PA alone for 24h. Scale bar = 200 μm.

To determinate the appropriate intervention concentration as well as the cytotoxic activity of 3-MA and 4-PBA in prefrontal cells, the viability and apoptotic rate were measured with different concentrations of inhibitors. The results of the pre-experiment are shown in Figure S2 and Figure S3. Based on our experimental design, that is, not only observed the role of inhibitors in PA-induced apoptosis, but also could not cause excessive toxic effects. We finally selected the concentration of 5 mM 4-PBA and 2.5 mM 3-mA for the follow-up experiments.


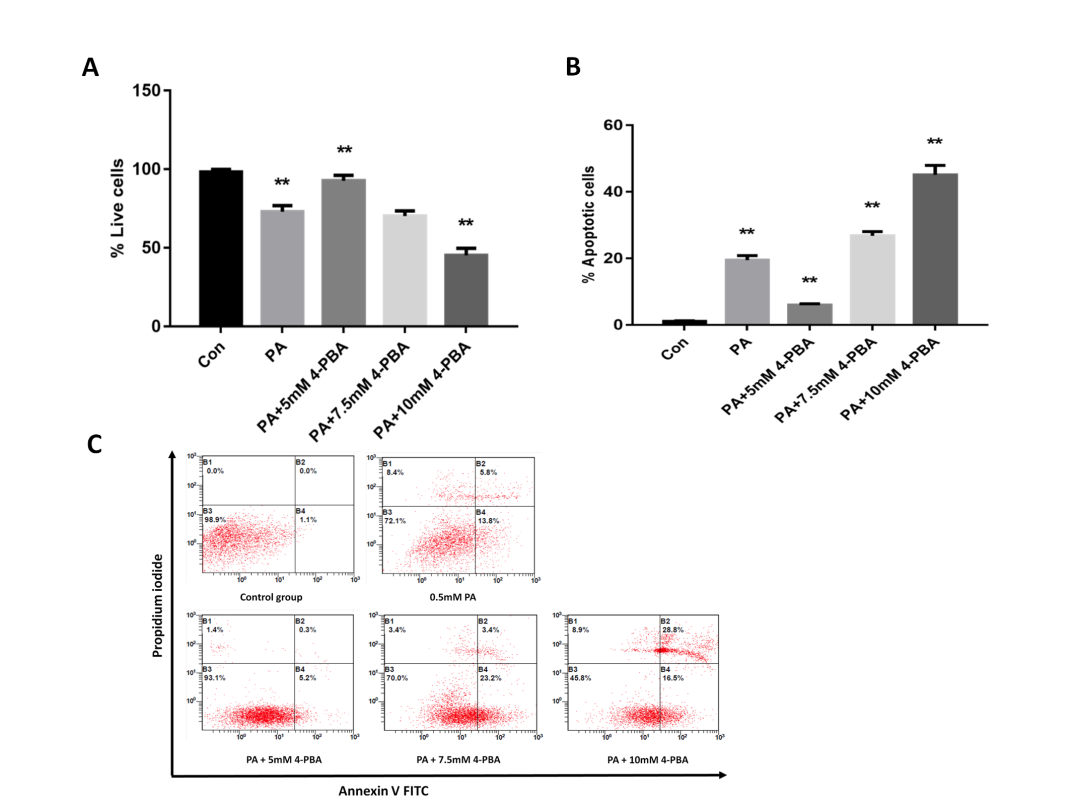


Figure S2. To explore treated concentration of 4-PBA inhibitor. (A) The effects of different concentrations of 4-PBA inhibitor on the viability rate of the prefrontal cells. (B) The effects of different concentrations of 4-PBA inhibitor on the apoptotic rate of the prefrontal cells. (C) Flow cytometry analysis was performed to measure the viability and apoptotic rate of the prefrontal cells. Data are presented as means ± SEM. * p < 0.05 versus control (Con).

**
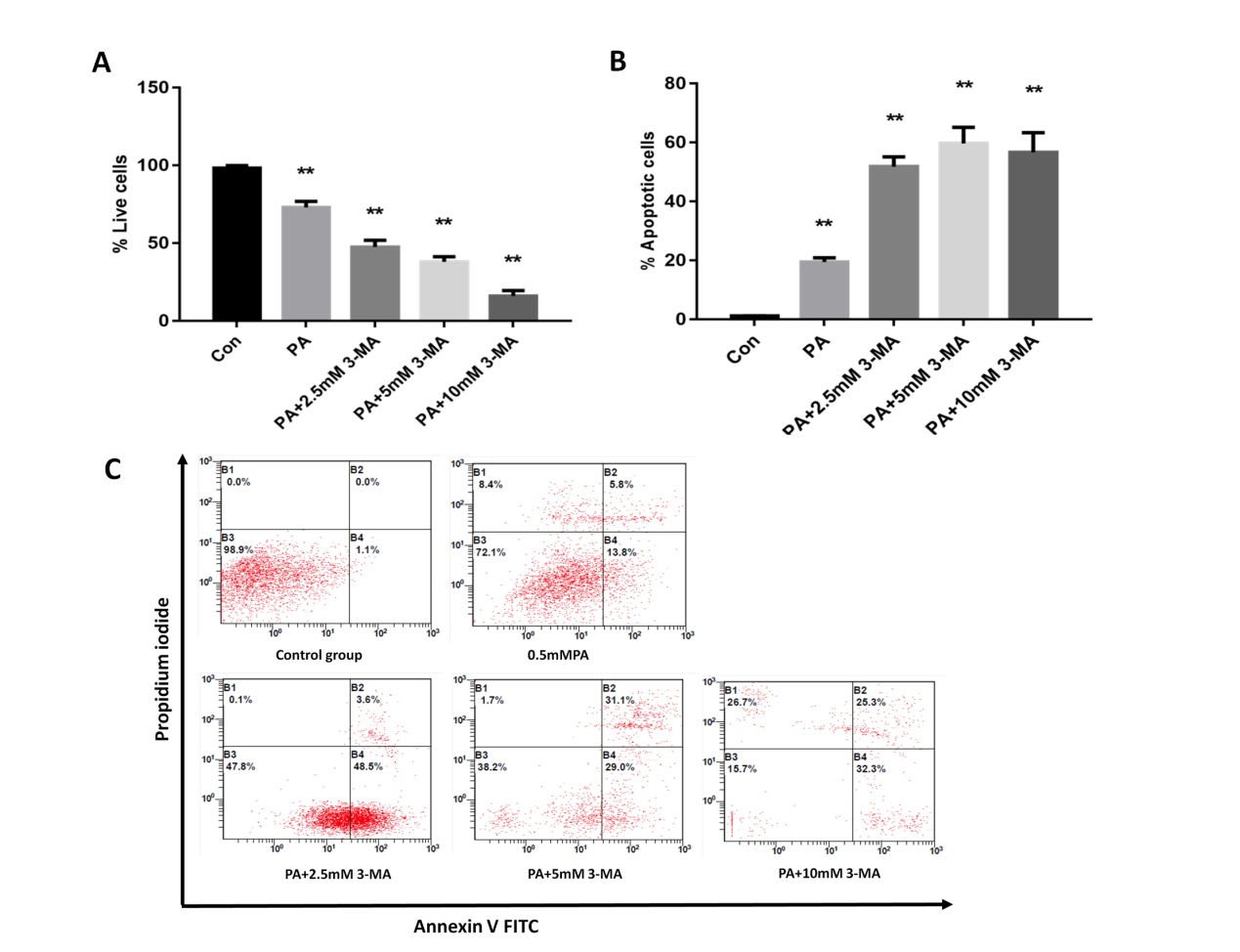
**

Figure S3. To explore treated concentration of 3-MA inhibitor. (A) The effects of different concentrations of 3-MA inhibitor on the viability rate of the prefrontal cells. (B) The effects of different concentrations of 3-MA inhibitor on the apoptotic rate of the prefrontal cells. (C) Flow cytometry analysis was performed to measure the viability and apoptotic rate of the prefrontal cells. Data are presented as means ±SEM. * p < 0.05 versus control (Con).

The amount of LC3 at a certain time point does not indicate autophagic flux, and therefore, it is important to measure the amount of LC3-II delivered to lysosomes by comparing LC3-II levels in the presence and absence of lysosomal protease inhibitors. As a lysosomotropic agent, Chloroquine (CQ), raises the lysosomal pH, blocking the fusion of autophagosomes with lysosomes, resulting in the accumulation of damaged autolysosomes. In this study, prefrontal cells were treated with 20 μM CQ (C6628, Sigma-Aldrich Corp.) compound with PA (0.5 mM) or PA alone for 24h, and then LC3 II levels were detected. We found that PA and CQ could significantly increase the autophagosome marker LC3-II/I (P＜0.01), indicating the accumulation of autophagosomes. The amount of LC3-II further accumulates in the presence of lysosomal protease inhibitors CQ compared to PA group, this would indicate enhancement of the autophagic flux.


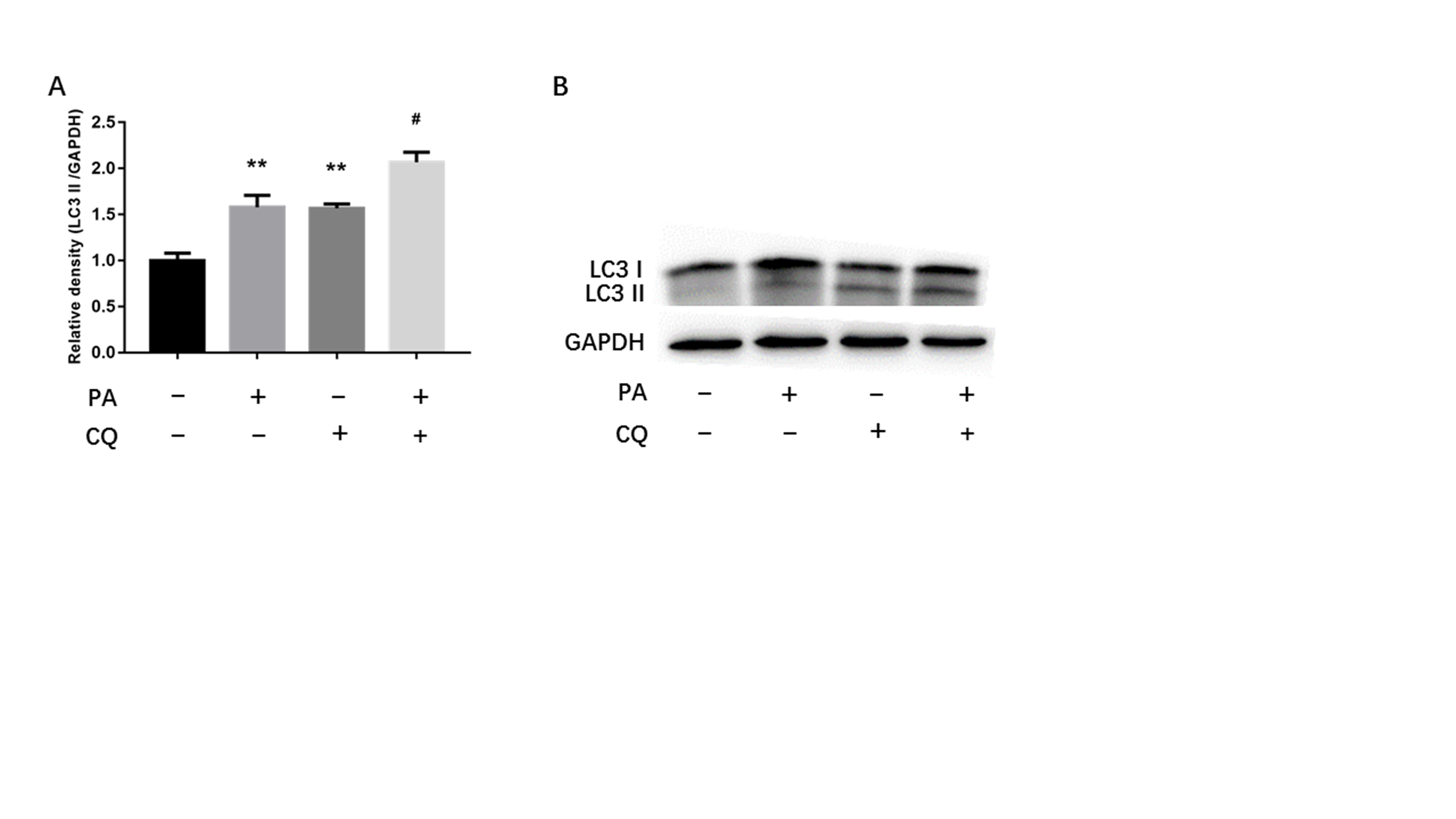


Fig. S4. Effects of CQ on the expression of autophagy markers in PA-treated prefrontal cells. The protein levels of LC3 (A) in prefrontal cells, and a representative Western blot image was shown (B). Data are presented as means ± SEM. ** p < 0.01 versus control (Con); # p < 0.05 versus PA.

The effect of 3-MA and 4-PBA alone is an important control. Therefore, we tested the effect of 4-PBA and 3-MA alone on the expression of apoptotic and ER stress related proteins using primary cultured prefrontal cells. There was no significant difference in apoptotic and ER stress related proteins expression between control group and autophagy inhibitor 3-MA group as well as control group and ER stress inhibitor 4-PBA group, as shown in Fig. S5 and S6 (P > 0.05).


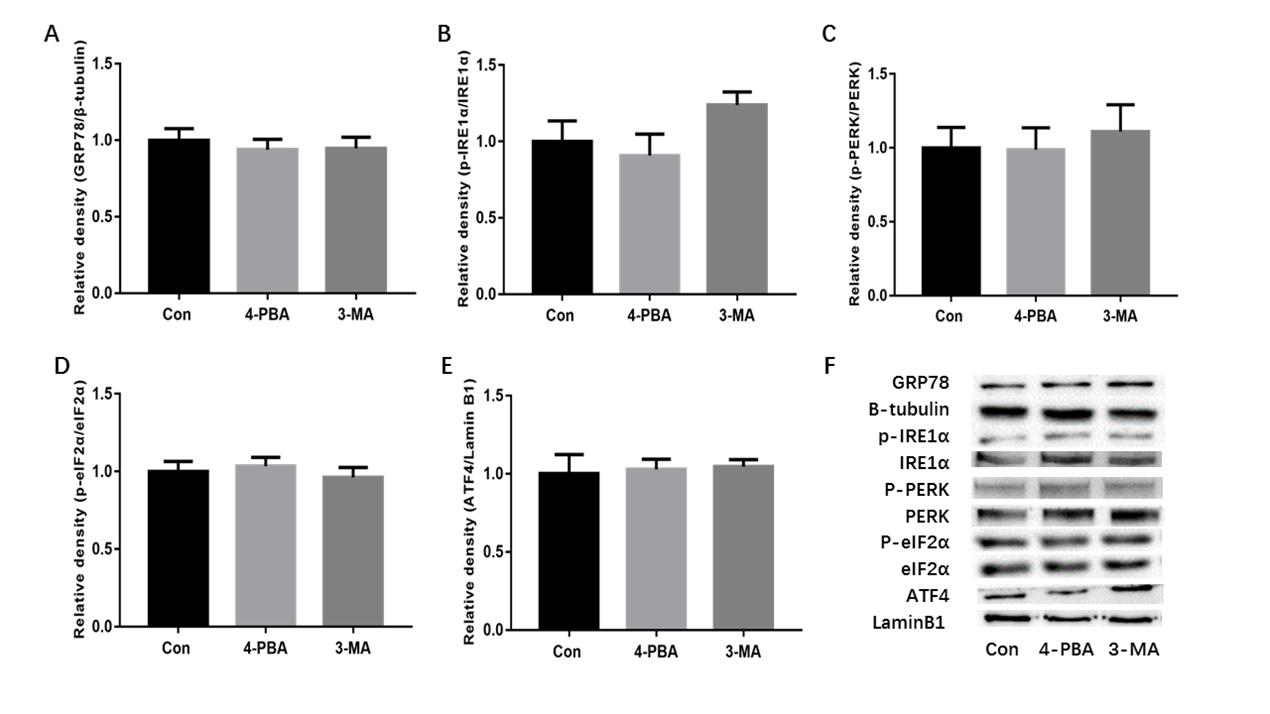


Fig. S5. Effects of 4-PBA and 3-MA alone on the expression of ER stress markers in prefrontal cells. The relative protein levels of GRP78 (A), p-IRE1α (B), p-PERK (C), p-eIF2α (D) and ATF4 (E) in prefrontal cells, and a representative Western blot image was shown (F). Data are presented as means ± SEM.


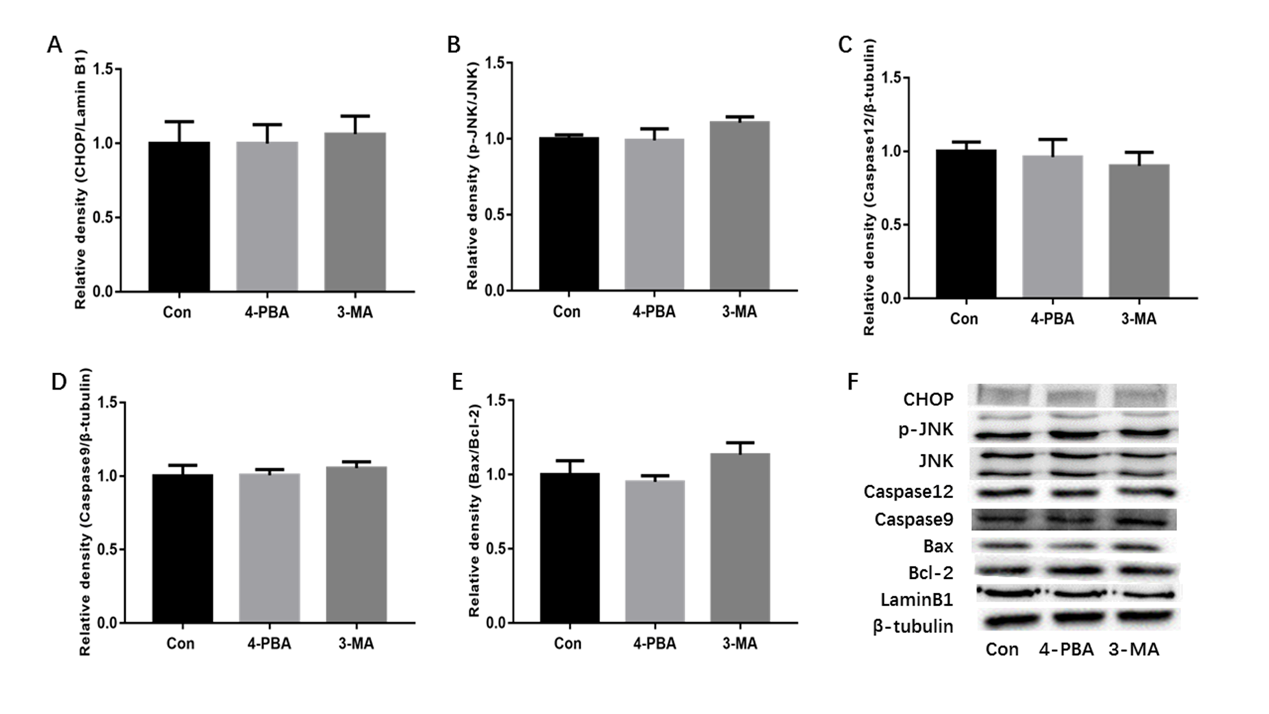


Fig. S6. Effects of 4-PBA and 3-MA alone on the expression of apoptosis markers in prefrontal cells. The protein levels of apoptosis proteins, including CHOP (A), p-JNK (B), caspase 12 (C), caspase 9 (D) and Bax/Bcl-2 (E) in prefrontal cells, and a representative Western blot image was shown (F). Data are presented as means ± SEM.
